# Emerging Topics and Trends in Neutrophil Extracellular Traps in ARDS: A Bibliometric and Visual Analysis

**DOI:** 10.1155/mi/1015955

**Published:** 2025-12-29

**Authors:** Yike Wang, Zixin Luo, Shaoyu Fu, Qianduo Li, Zhiyuan Zhang, Nan Huang, Kang Zou, Lingyan Zou

**Affiliations:** ^1^ The First Clinical Medical College, Gannan Medical University, Ganzhou, 341000, Jiangxi Province, China, gmu.cn; ^2^ Department of Critical Care Medicine, The First Affiliated Hospital of Gannan Medical University, Ganzhou, 341000, Jiangxi Province, China, gmu.cn; ^3^ Department of Respiratory and Critical Care Medicine, Ganzhou Hospital-Nanfang Hospital, Southern Medical University (Ganzhou People’s Hospital), Ganzhou, 341000, Jiangxi Province, China, fimmu.com

**Keywords:** ARDS, CiteSpace, NETs, VOSviewer, WoSCC

## Abstract

**Background:**

Acute respiratory distress syndrome (ARDS) is a critical illness with significant impacts on human respiratory health. Neutrophil extracellular traps (NETs), secreted by neutrophils, play dual roles in immune defense and tissue inflammation. Recent studies have highlighted the association between NETs and ARDS, yet no comprehensive bibliometric analysis has been conducted in this field. This study aims to analyze the research progress and evolving hot spots related to NETs in ARDS through bibliometric methods.

**Methods:**

Articles published between 2011 and 2024 were retrieved from the Web of Science Core Collection (WoSCC), PubMed, and Embase. Data analysis was performed using visualization tools such as VOSviewer, CiteSpace, and Microsoft Office Excel 2021.

**Results:**

A total of 328 articles were analyzed. The annual publication trend in this field has shown a steady increase, with China and the United States leading in research output. Frontiers in Immunology stands out for its high publication volume and citation count, indicating strong reference value. The most prolific author is Zhang, Hao from Zhongshan Hospital, Fudan University, while Egeblad, Mikala from Cold Spring Harbor Laboratory holds the highest citation count. Inflammatory response is a research focus in this field, and the association between NETs and thrombus formation represents an emerging research hotspot within the domain. Research and development of NETs–targeted therapies for acute lung injury (ALI) and ARDS is an important direction for future research.

**Conclusion:**

This bibliometric analysis comprehensively summarizes research progress and hotspot evolution in NETs–related ARDS studies, providing valuable insights for researchers and inspiring future research directions.

## 1. Introduction

Acute lung injury (ALI) is an important cause for the development of acute respiratory distress syndrome (ARDS), which is a severe clinical condition associated with high incidence and mortality rates, posing a significant challenge to critical care medicine. ARDS is an acute onset of hypoxemia and bilateral pulmonary edema due to excessive alveolar‐capillary permeability [1]. Over the past few years, a growing number of studies have emphasized the potential key role of neutrophil extracellular traps (NETs) in the pathogenesis of ALI/ARDS, making them a prospective target for therapeutic endeavors [2]. Neutrophils play a crucial role as a fundamental component of the host immune system, primarily participating in the immune defense process against a variety of microorganisms through three mechanisms: degranulation, phagocytosis, and the formation of NETs [3]. Among these, NETs have complex functions. They participate in immune defense, but can also cause tissue inflammation. During the year 2004, Brinkmann et al. [4] discovered, through transmission electron microscopy, that activated neutrophils produce significant extracellular fibrous structures. They further analyzed using immunofluorescence techniques and found that neutrophil elastase forms spherical structures, while H2A‐H2B histones + DNA form chromatin structures that appear as bundles, combining to form a complex mesh‐like structure known as NETs [4]. In the context of the host’s defense against bacterial infections, NETs are of indispensable importance. But once they are overactivated, tissue damage occurs, transforming them into what can be likened to a “double‐edged sword.” An increasing number of researchers have found that NETs are linked to a variety of inflammatory diseases, among which are ALI/ARDS.

While research on the mechanism of action and regulation of NETs in ALI/ARDS remains in the exploratory stage, phased progress has been achieved in three core directions currently focused on NETs: the identification of therapeutic targets, the development of therapeutic strategies, and the application of biomarkers. First, in the field of therapeutic target identification, studies have confirmed that inhibiting the cGAS‐STING pathway or platelet glycoprotein VI (GPVI) can significantly alleviate NETs–mediated ALI, suggesting that these may serve as novel therapeutic targets for ALI/ARDS [5, 6]. As a key molecule in NET formation, peptidylarginine deiminase 4 (PAD4) is also regarded as a potential therapeutic target [7]. Zhao et al. [8] found that PAD4 expression is upregulated in LPS‐induced ALI, and its selective inhibitor, Thr‐Asp‐F‐amidine (TDFA), can significantly reduce pulmonary edema, inflammatory infiltration, and oxidative stress, protect the integrity of the alveolar epithelial barrier, and improve survival rates. A study by Wu et al. [9] confirmed that *Bletilla striata* polysaccharide can regulate the PAD4 pathway, reduce NET levels, alleviate NET‐induced pyroptosis of alveolar macrophages, and significantly mitigate ARDS‐related lung injury [9]. Second, based on the identified targets, the development of therapeutic strategies has gradually advanced and demonstrated potential value. For instance, Huang et al. [10] developed cationic nanoparticles that can effectively neutralize cell‐free DNA (cfDNA), inhibit the activation of the cGAS–STING pathway, and significantly alleviate lung injury [10]. Zhao et al. [11] constructed macrophage membrane‐coated nanomedicines that can significantly downregulate PAD4 expression, inhibit NET formation, and promote M2 macrophage polarization, thereby effectively reducing ALI [11]. Xie et al. [12] found that Taohe Chengqi Decoction can significantly alleviate sepsis‐induced ALI by inhibiting PAD4–mediated NET formation [12]. Additionally, in terms of biomarker application, NETs have shown significant potential for clinical translation. Data indicate that in bacterial infection‐induced ALI models and ARDS patients, NET levels in the alveoli and plasma are significantly elevated, and this elevation is closely associated with the severity of lung injury and mortality [13]. This association makes NETs a promising potential biomarker for ALI/ARDS, which can be used for disease assessment and prognosis judgment. Hence, it holds significant importance to carry out a quantitative analysis regarding the research advancement, growth outcomes, and future outlook of NETs in ALI/ARDS.

Bibliometrics is a quantitative analytical method that integrates library and information science with various disciplines [14]. Its core advantage lies in systematically analyzing the knowledge structure and development laws of research fields through objective data. Compared with the limitation of traditional reviews, which mostly rely on researchers’ subjective judgments to summarize progress, this method can reveal macrolevel characteristics such as the disciplinary core and the evolution of research hot spots in a systematic and quantitative manner [15]. Currently, researchers have published bibliometric articles related to NETs or ALI/ARDS. For example, Xiao et al. [16] applied bibliometric methods to map the knowledge network between NETs and tumors, revealing that NETs are promising anti‐tumor targets. Muñoz‐Caro et al. [17] systematically analyzed the research progress of NETs induced by protozoa and helminth parasites from 2008 to 2024 via bibliometric analysis, pointing out that therapeutic targeting of NETs and comparative studies on different hosts and parasite species are emerging trends in this field. Zhou et al. [18] used bibliometric methods to systematically analyze the research progress of mesenchymal stem cell‐derived exosomes (MSC‐exosomes) in ALI/ARDS from 2013 to 2022, indicating that exploring the potential mechanism of MSC‐exosomes in treating ALI/ARDS is the research hotspot. In addition, our team has previously conducted a series of bibliometric studies focusing on the top 100 most cited high‐impact literatures in the field of ALI/ARDS [19], the association between ALI/ARDS and skin barrier dysfunction [20], as well as the interactions between ALI/ARDS and ferroptosis and pyroptosis [21]. However, there is currently no one conducting bibliometric analysis on NETs in ALI/ARDS, and the related research hot spots and trends are unclear. Therefore, in the present research, we utilized analysis based on bibliometrics to examine the research directions and hotspot evolution of NETs in ALI/ARDS from 2011 to 2024, thereby constructing a knowledge system chart of this field.

## 2. Materials and Methods

### 2.1. Data Resources and Retrieval Strategies

**Figure 1:**
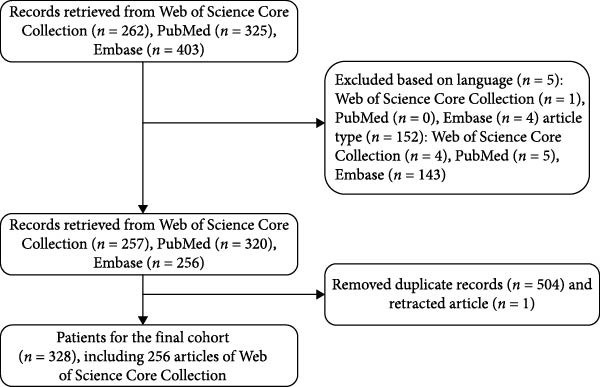
Article retrieval flow chart.

Web of Science (WOS) has become a key tool for bibliometric analysis due to its rigorous selection criteria, comprehensive citation network, and robust citation analysis capabilities [22]. However, its coverage focuses on high‐impact journals, with relatively limited inclusion of clinical trials and drug‐related articles. In contrast, PubMed comprehensively covers literature in the biomedical field, featuring timely updates and a broad scope of inclusion [23]. Embase, meanwhile, offers extensive coverage of pharmaceuticals, medical devices, toxicology, and European journals, serving as a valuable supplement to WOS and PubMed [24]. On April 5, 2025, we searched for relevant literature published from January 1, 2011 to December 31, 2024, in the WOS Core Collection (WoSCC), PubMed, and Embase databases based on titles and abstracts. The retrieval methods are as follows: #1: “acute respiratory distress syndrome” OR “ARDS” OR “acute lung injury” OR “ALI” #2: “Neutrophil Extracellular Traps” OR “NETs” OR “NETosis” #3: #1 AND #2. We set the publication language to “English,” and the document types to “Article” and “Review,” after which we imported the articles into EndNote X9 to eliminate duplicate records and perform screening. Through these operations, we ultimately identified 328 articles (Figure 1).

### 2.2. Data Analysis

In this study, bibliometric analysis software including CiteSpace (Version 6.4 R1), VOSviewer (Version 1.6.20), and Bibliometrix (based on R‐4.4.1) was employed to process and visually analyze the included literature data. In addition, Microsoft Office Excel 2021 was used to generate bar charts and line charts of annual publication output. VOSviewer, a network data visualization software, provides intuitive views reflecting the weights and connections of analyzed data [25]. Within the views yielded by VOSviewer, nodes signify authors, keywords as well as other elements. The size and color of the nodes represent the quantity and categorization of these items, respectively. The thickness of the lines between nodes reflects the degree of collaboration or co‐citation of the items. In order to reveal the major research forces and scientific research collaboration patterns, we used VOSviewer to conduct collaboration analysis on countries, institutions, authors, and journals, respectively. To identify research hot spots and thematic structures, we leveraged VOSviewer for keyword co‐occurrence analysis. For the purpose of recognizing the key knowledge foundations in the field, we employed VOSviewer to perform co‐cited reference clustering analysis. By resorting to visual approaches, CiteSpace uncovers the structure, regularities and distribution of scientific knowledge [26]. Thus, we utilized CiteSpace to conduct keyword burst analysis and reference burst analysis and generated a keyword timeline map, so as to reveal emerging research hot spots and the evolutionary trajectory of research topics. Additionally, we used Bibliometrix to perform frequency statistics on the number of publications by countries, institutions, authors, and journals, aiming to quantify the core contributors in the field. Owing to the absence of references in the articles exported from PubMed and Embase, we utilized 256 articles exported from WOS to conduct citation‐related analysis.

## 3. Results

### 3.1. The Quantitative Analysis Targeting Publications

**Figure 2:**
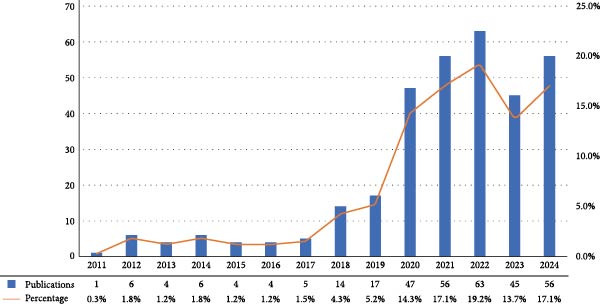
The number of papers released on NETs in ALI/ARDS globally between 2011 and 2024.

Figure 2 illustrates the publication trend of research on NETs in ALI/ARDS from 2011 to 2024. Overall, there has been a rapid increase in annual publications within this field. From one article in 2011 to 56 in 2024, it is evident that the area is gaining increasing attention from scholars. According to Figure 2, we can divide the entire period into three phases. The first phase spans from 2011 to 2017, during which there was a relatively low number of publications, indicating that the research was in its nascent stage. The second phase extends from 2018 to 2022, with a significant increase in the number of publications, peaking at 63 articles published in 2022. The third phase covers the years 2023–2024, during which there was a slight decrease in the number of publications, but the volume remained high, suggesting that research in this area continues to evolve.

### 3.2. Detailed Analysis Targeting Countries and Institutions

With the aim of investigating which countries have made substantial contributions to the study of NETs in ALI/ARDS, we analyzed the relevant countries and institutions via VOSviewer (Figure 3) and summarized and ranked the top 10 countries and institutions by publication volume (Table 1). As shown in Table 1, China tops the list in terms of the number of publications, succeeded by the United States. Figure 3A shows that the cooperation between the United States and China is very close, and these two countries also frequently collaborate with other countries, such as Germany, the United Kingdom, and France. In terms of institutions, we found that the top 10 institutions are all from China or the United States. Among them, Fudan University in China (*n* = 12) has the highest publication volume and plays a leading role in this field. The University of Michigan in the USA closely follows with nine articles. Subsequently, we set the minimum publication volume for each institution to two and conducted a visual analysis of these institutions (Figure 3B). We found that most institutions mainly collaborate with domestic institutions. For example, Fudan University collaborates very closely with Central South University and Southern Medical University, but has less collaboration with institutions from other countries.

Figure 3Countries (A) and institutions (B) cooperation cluster view of studies concerning NETs in ALI/ARDS. The magnitude of the nodes stands for the publication volume, while the thickness of the lines concerning the nodes demonstrates the intensity of cooperation between two nodes.

**Figure:**
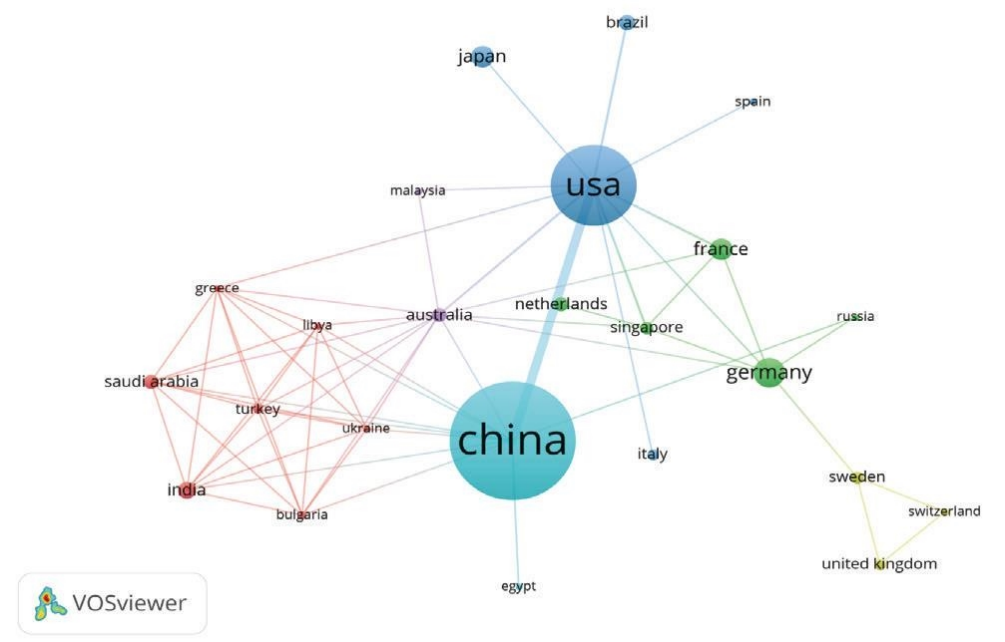
(A)

**Figure:**
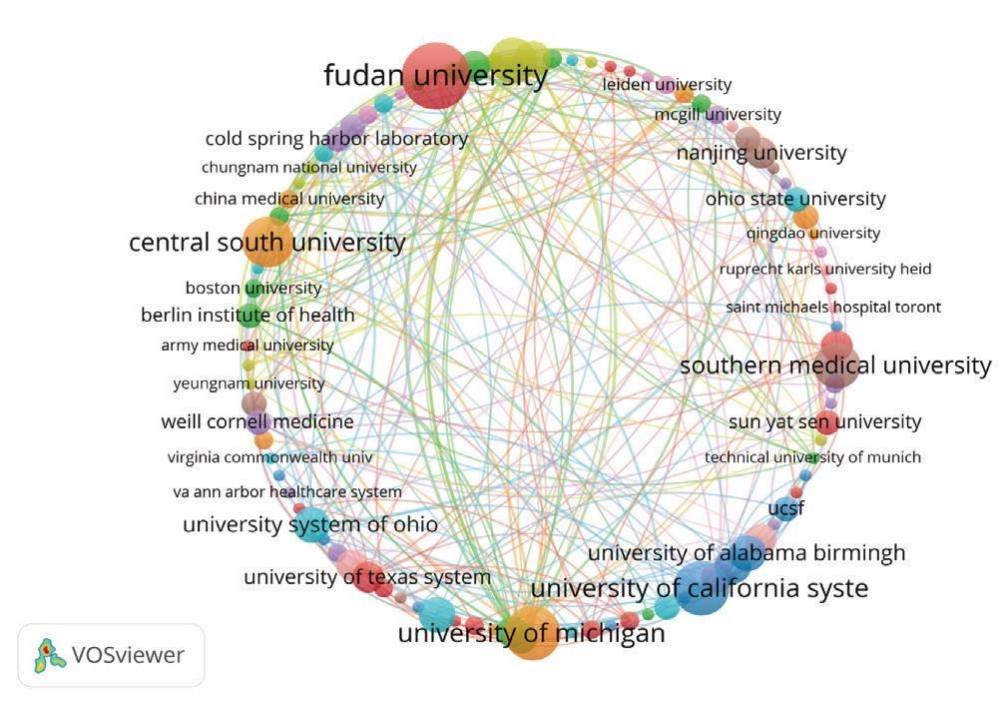
(B)

**Table 1:** Comprehensive analysis of the 10 countries and institutions at the top.

Rank	Country	Publications	Total link strength	Affiliations	Publications	Total link strength
1	China	100	177	Fudan University (China)	12	87
2	USA	68	181	University of Michigan (USA)	9	72
3	Germany	18	39	Central South University (China)	8	51
4	France	12	36	Harvard University (USA)	7	76
5	Japan	12	5	University of California System (USA)	7	25
6	India	9	11	Southern Medical University (China)	7	22
7	Brazil	8	18	University of Alabama Birmingham (USA)	5	29
8	Australia	7	15	Zhejiang University (China)	5	22
9	Netherlands	7	14	University of Pittsburgh (USA)	5	16
10	Canada	5	10	Shandong University (China)	5	12

### 3.3. Detailed Analysis Targeting Top Journals and Co‐Cited Journals

Studies associated with NETs in ALI/ARDS have been published across 187 journals. Table 2 shows 15 journals with the most publications and 15 journals with the highest co‐citations. Frontiers in Immunology serves as the journal with the most publications (*n* = 25) and simultaneously as the journal with the most co‐citations (*n* = 615). Furthermore, as illustrated in Supporting Information 1: Figure S1, articles from Frontiers in Immunology have a very close relationship with articles from other journals, indicating that Frontiers in Immunology has made a significant contribution to the field. Among the 15 journals that lead the pack in citation counts, four articles have witnessed the accumulation of citations exceeding 400 times. The impact factors of these highly cited journals are generally very high, with the highest being The Lancet (IF = 98.4), indicating that the authority of these journals is widely recognized by scholars, and many researchers are more likely to cite articles from these journals.

**Table 2:** Comprehensive analysis of the 15 journals and co‐cited journals at the top.

Rank	Journal	Count	IF (2023)	JCR (2023)	Co‐cited iournal	Co‐citation	IF (2023)	JCR (2023)
1	Frontiers in Immunology	25	5.7	Q1	Frontiers in Immunology	615	5.7	Q1
2	Jci insight	10	6.3	Q1	Blood	560	21.0	Q1
3	International Immunopharmacology	7	4.8	Q1	Journal of Immunology	455	3.6	Q2
4	International Journal of Molecular Sciences	7	4.9	Q2	Journal of Clinical Investigation	401	13.3	Q1
5	Blood	6	21.0	Q1	American Journal of Respiratory and Critical Care Medicine	373	19.3	Q1
6	Scientific Reports	6	3.8	Q1	Nature Medicine	356	58.7	Q1
7	American Journal of Physiology‐lung Cellular and Molecular Physiology	4	3.6	Q1	Plos One	352	2.9	Q1
8	Frontiers in Pharmacology	4	4.4	Q1	New England Journal of Medicine	345	96.2	Q1
9	Inflammation	4	4.5	Q2	Science	303	44.7	Q1
10	Journal of Clinical Investigation	4	13.3	Q1	Nature Reviews Immunology	285	67.7	Q1
11	Plos One	4	2.9	Q1	Journal of Experimental Medicine	271	12.6	Q1
12	Shock	4	2.7	Q1	Proceedings of the National Academy of Sciences of the United States of America	251	9.4	Q1
13	Biomolecules	3	4.8	Q1	Jci Insight	243	6.3	Q1
14	Life Sciences	3	5.2	Q1	Scientific Reports	232	3.8	Q1
15	Mediators of Inflammation	3	4.4	Q2	Lancet	227	98.4	Q1

Abbreviations: IF, impact factor; JCR, journal citation reports.

### 3.4. Detailed Analysis Targeting Top Authors and Co‐Cited Authors

The authors ranking in the top 10 are presented in Table 3. It is evident from Table 3 that ZHANG HAO from Fudan University has the highest number of publications. It was observed that LI and YI collaborates closely with other authors, while most other authors primarily collaborate with authors from their own institutions (Supporting Information 2: Figure S2). It is hoped that all authors can strengthen their cooperation to promote the development of the field and make collective progress. Table 3 also displays the top 10 most‐cited authors. EGEBLAD and MIKALA, from Cold Spring Harbor Laboratory, Cancer Center, Cold Spring Harbor, NY, has the maximum number of citations (*n* = 212), with his primary research interests lying in oncology and immunology.

**Table 3:** Comprehensive analysis of the 10 authors and co‐cited authors at the top.

Rank	Authors	Count	Co‐cited authors	Local citations
1	Hao Zhang	9	Mikala Egeblad	212
2	Yi Li	8	Mark R. Looney	186
3	Mark R. Looney	8	Jason S. Knight	138
4	Qian Li	7	Yu Zuo	138
5	Ying Zhang	7	Betsy Barnes J.	136
6	Mikala Egeblad	7	Andrew Weber	136
7	Haitao Li	6	Amelia Baxter‐Stoltzfus	133
8	Pinhua Pan	6	Massimo Loda	133
9	Changhong Miao	6	Minhui Dai	88
10	Minhui Dai	6	Haitao Li	88

### 3.5. Detailed Analysis Targeting Co‐Cited References and Bursts in the Citations of References

Whenever a publication finds itself being cited by two or more other publications in tandem, it assumes the identity of a co‐cited reference [27]. The quantity of co‐citations serves as a manifestation of the significance of a document within a specific research domain. From 2011 to 2024, there were 12,200 co‐cited references studying the relationship between NETs and ALI/ARDS. Supporting Information 3: Table S1 lists the top 10 co‐cited references. Subsequently, we set the minimum number of co‐citations to 20, resulting in 38 references, from which we created a co‐citation cluster view of references (Supporting Information 4: Figure S3). The document written by Brinkmann et al. [4], which first identified NETs, has been cited jointly as many as 129 times.

A citation burst refers to the frequent citation of documents by scholars within a specific domain over the course of a period. It is demonstrated in Supporting Information 5: Figure S4 that the top 10 references which have the strongest citation bursts, with the main research summarized in Supporting Information 3: Table S2. A reference burst diagram can reflect the documents with significant influence with the passage of time; the red line indicates the year when the reference had a high volume of citations. From the diagram, one can read the citation intensity of the references, the time of appearance or cessation, and the average year of emergence. The earliest emergence of citation bursts concerning references came into view in 2014, and the reference with the longest duration of bursts is Caudrillier et al. [28], which remained in a burst period from 2014 to 2017, lasting for 3 years (Supporting Information 5: Figure S4).

### 3.6. Detailed Analysis of Hot Spots and Frontiers

Through co‐occurrence analysis of keywords, it is possible to quickly identify research hot spots in NETs within ALI/ARDS. By setting the minimum occurrence threshold to 20, a total of 30 keywords were selected. Based on the frequency of keyword occurrence, the top five keywords with the highest frequency are NETs, inflammation, ALI, immunology, and COVID‐19 (Figure 4A). According to the keyword co‐occurrence clustering view, three clusters were formed (Figure 4B). The main keywords in the red cluster include “neutrophil extracellular traps,” “inflammation,” “acute lung injury,” and “neutrophil.” The main keywords in the blue cluster include “immunology,” “thrombosis,” “activation,” and “sepsis,” The main keywords in the green cluster include “COVID‐19,” “neutrophils,” “nets,” and “netosis.”

Figure 4Keywords co‐occurrence density view (A) and cluster view (B).

**Figure:**
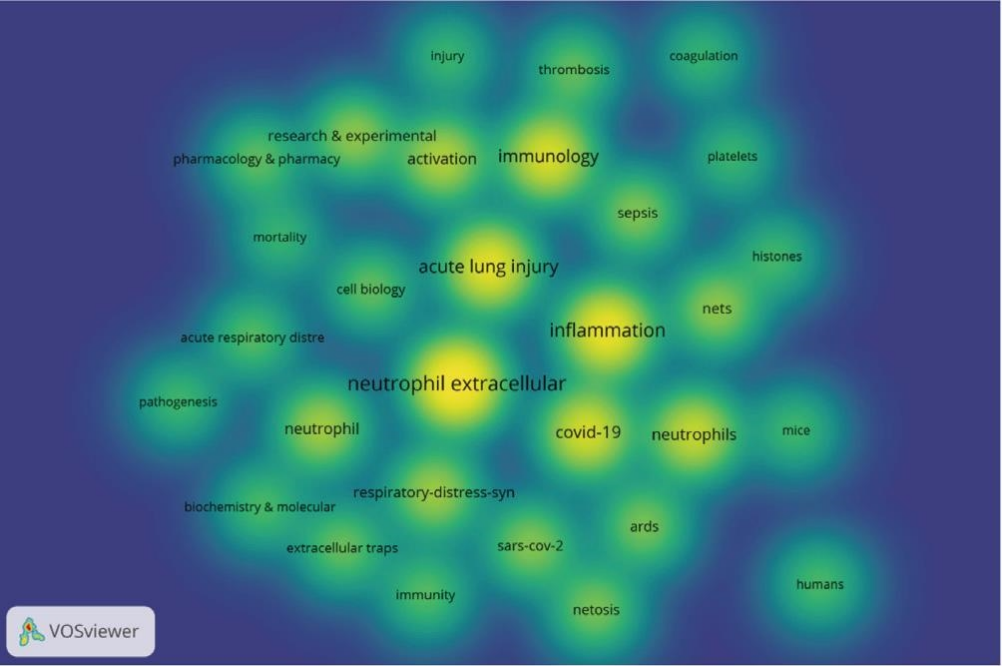
(A)

**Figure:**
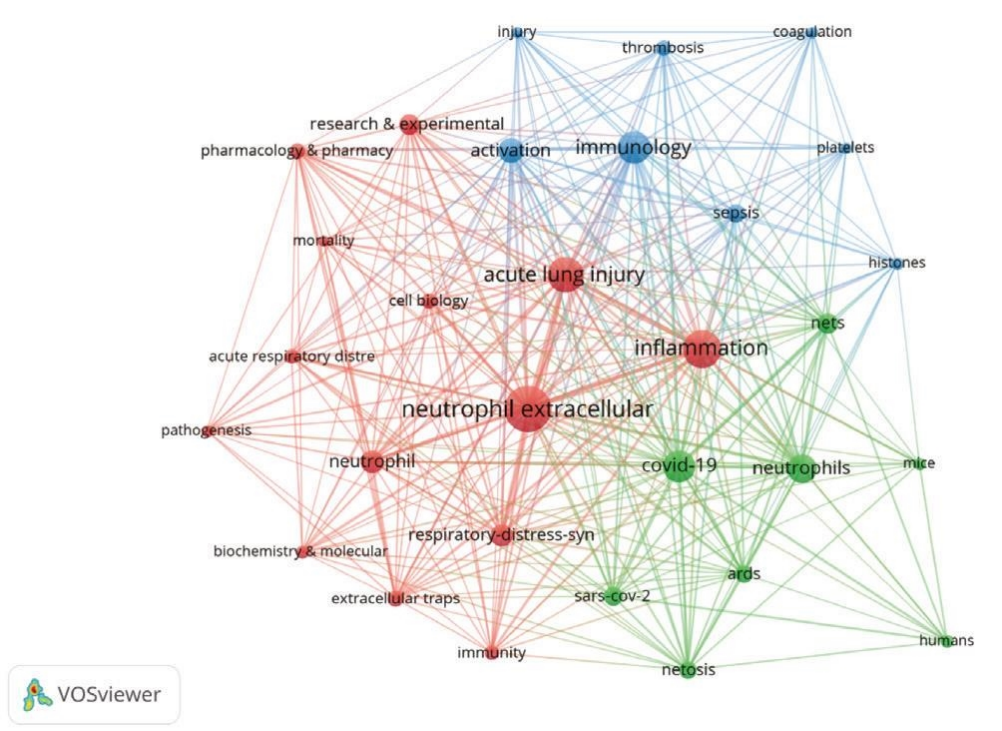
(B)

To gain a deeper understanding of the research topics actively discussed during a specific period in this field, we further utilized the Bursts analysis function of CiteSpace, with the results shown in Figure 5A. Due to the outbreak of the COVID‐19 pandemic, “coronavirus disease 19” experienced a brief surge in 2020 and 2021. Additionally, it is worth noting that “thrombosis” and “recruitment” began to show a burst in 2021 and 2022, respectively. Figure 5B is a timeline chart created using CiteSpace, where CiteSpace arranges keywords on the horizontal axis according to time, and displays the co‐occurrence relationships between keywords and their evolution over time through lines and nodes. As can be seen from Figure 5, overtime, research topics have gradually shifted from basic research‐related fields such as biochemistry and molecular biology and pharmacology and pharmacy to diseases including ARDS and coronavirus disease 2019 (Figure 5B).

Figure 5Top 10 strong citation outbreaks of keywords (A). The timeline chart of keyword co‐occurrence clustering (B).

**Figure:**
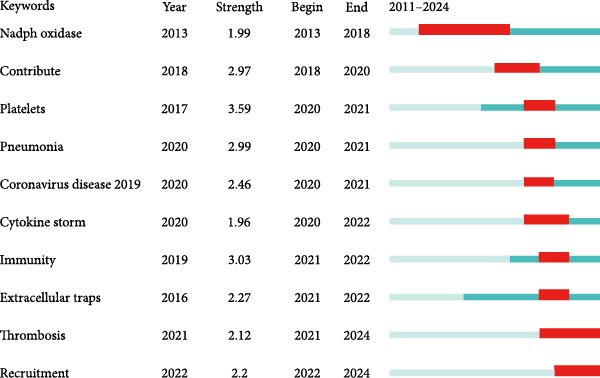
(A)

**Figure:**
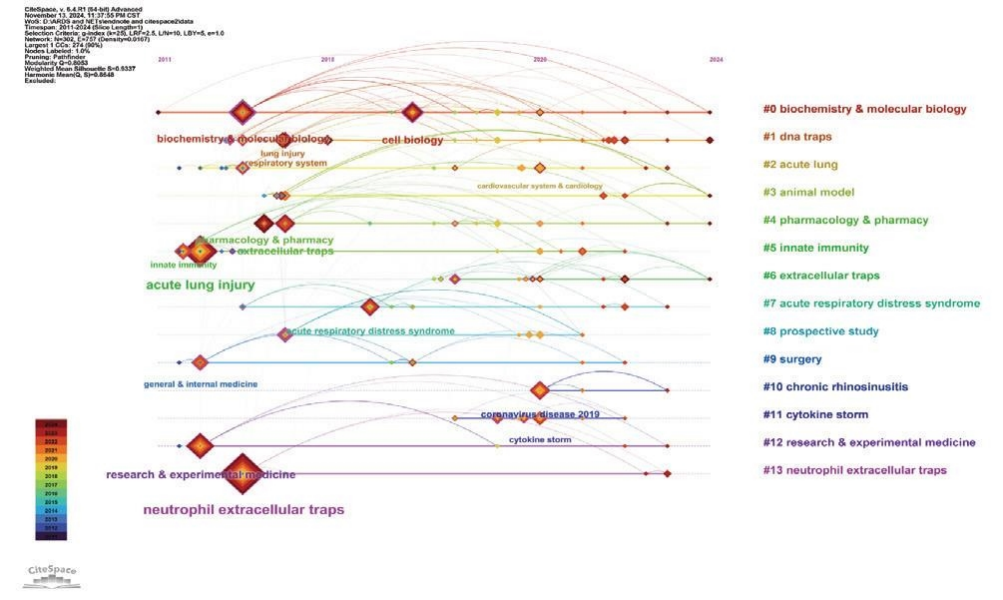
(B)

## 4. Discussion

### 4.1. General Information

From 2011 to 2024, the annual number of publications in this field generally demonstrated an upward tendency. The surge in publications in 2020 could be attributed to the emergency of the COVID‐19 pandemic, which led to a proliferation of articles related to COVID‐19. However, starting from 2020, the yearly output publications exceeded 40, indicating that although the research concerning NETs in ALI/ARDS remains at an early phase, it nonetheless possesses significant potential for further development. China and the United States are at the forefront in this field, with the top 10 institutions in terms of publication output all being from either China or the United States. Moreover, academic cooperation between the two countries is also very close. This indicates that breaking down academic barriers and strengthening international cooperation can broaden the research horizons of both parties and promote the evolution of the scientific field. From the perspective of scientific research by authors, EGEBLAD and MIKALA from Cold Spring Harbor Laboratory has the highest number of citations (*n* = 212), providing reliable reference value for scholars in the field. He linked abnormal NETs formation to pulmonary diseases, thrombosis, airway mucus secretion, and cytokine production, proposed that NETs‐containing microthrombi with neutrophil–platelet infiltration cause immune thrombosis in ARDS and discovered that disulfiram, an FDA‐approved drug for alcohol use disorder, can prevent NETs formation [29–31]. Additionally, T. Narasaraju is the founder and pioneer in this field. In 2011, he published the article “excessive neutrophils and neutrophil extracellular traps contribute to acute lung injury in influenza pneumonia” for the first time, demonstrating that the formation of NETs in influenza pneumonia is induced by redox enzymes [32].

### 4.2. Co‐Citation Network Analysis

By means of co‐citation analysis (Supporting Information 4: Figure S3), we are able to get an understanding of the research foundation of NETs in ALI/ARDS [26]. In 2004, Brinkmann et al. [4] from the Max Planck Institute for Infection Biology published an article titled “neutrophil extracellular traps kill bacteria,” which first revealed the existence of NETs [33]. The formation of NETs can be divided into two mechanisms: suicidal NETosis and vital NETosis. Suicidal NETosis, a common pathway for NETS formation, typically occurs when PMA activates protein kinase C (PKC) and the Raf‐MEK‐ERK signaling pathway, stimulating the activation of NADPH oxidase (NOX) to produce a large amount of reactive oxygen species (ROS) [34]. ROS act as signaling molecules, activating protein kinases, leading to the dissolution of the neutrophil’s nuclear membrane and decondensation of chromatin, thereby promoting the formation of NETs [34]. Vital NETosis refers to the production of mitochondrial NETs containing mitochondria but no nuclear DNA by living neutrophils after being triggered by granulocyte/macrophage colony‐stimulating factor (GM‐CSF) and subsequent short‐term toll‐like receptor 4 (TLR4) or complement factor 5a (C5a) receptor stimulation [35].

### 4.3. Research Hot Spots

Keyword co‐occurrence analysis (Figure 4B) and keyword burst analysis (Figure 5A) enable us to grasp the research hot spots of NETs in ALI/ARDS. Within the keyword co‐occurrence analysis (Figure 4B), “inflammation” serves as the core keyword of the biggest cluster (red cluster), suggesting that the inflammatory response constitutes a primary research hotspot in this domain. Keyword burst analysis (Figure 5A) reveals that “thrombosis” began to burst in 2021 and continued through 2024, demonstrating that thrombosis is an emerging research hotspot in this field.

#### 4.3.1. NETs are Amplifiers of the Inflammatory Response

ARDS is a life‐threatening inflammatory disease [36]. Despite the continuous advancement of critical care technology and organ support measures in recent years, there is still a lack of effective treatment regimens for ARDS in clinical practice, making it a major healthcare and socioeconomic burden worldwide [37]. Neutrophils are of crucial significance in the overwhelming inflammatory process of ALI that leads to ARDS [38]. Neutrophils can uncontrollably release NETs during sustained inflammation and excessive NETs can exacerbate the inflammatory response [39]. Macrophages, which act as key immune effector cells in lung tissue, are categorized into two subtypes by functional phenotypes: M1‐type, which exerts pro‐inflammatory properties, and M2‐type, which performs anti‐inflammatory roles [40]. The balance between these two types is crucial for maintaining immune homeostasis in lung tissue. Studies have confirmed that NETs can exacerbate the inflammatory response in ALI/ARDS by promoting the polarization of alveolar macrophages toward the M1 type [41]. In addition to regulating macrophage polarization, NETs can also exacerbate the inflammatory response by releasing key effector molecules. Among these, high‐mobility group box 1(HMGB1) of NET origin serves as a crucial pro‐inflammatory mediator [42]. By binding to receptor for advanced glycation end products (RAGE), it stimulates the dynein‐dependent signaling pathway, further triggering molecular cascades within macrophages: lysosomal rupture releases cathepsin B (CatB), followed by inflammasome assembly and caspase‐1 activation, which ultimately induces macrophage pyroptosis, and thus, aggravates the inflammatory response [42].

#### 4.3.2. The Relationship Between NETs and Thrombosis

Thrombosis refers to a pathological process in which blood coagulates abnormally within blood vessels and forms blood clots, obstructing the vascular lumen [43]. This process can lead to insufficient blood perfusion in local tissues, thereby causing organ damage or even death [43]. Studies have shown that NETs provide a scaffold and stimulus for thrombus formation, facilitate the deposition of von Willebrand factor (VWF), fibrinogen, and fibrin, forming a positive feedback activation of coagulation [44]. Furthermore, NETs take polymorphonuclear neutrophils (PMNs) as their core component [45]. Uncontrolled activation of PMNs leads to the release of large amounts of ROSand various proteases, which damage tissue cells and vascular endothelial cells, induce platelet adhesion, activation and aggregation, and thus, promote thrombus formation [7]. Consequently, the potential mechanisms of NETs in immunothrombosis of ALI/ARDS have aroused great interest among researchers, and many are conducting in‐depth studies. Subsequently, researchers found that NETs can enhance the aggregation capacity of platelets in pulmonary blood vessels and induce microthrombus formation, thereby causing alveolar congestion and impairment of alveolar epithelial barrier function [45, 46]. In addition, another study analyzed lung autopsy samples from patients with COVID‐19‐associated ARDS and found that microthrombi containing NETs were significantly associated with neutrophil–platelet infiltration in lung tissue, which further confirmed the pathological significance of NETs in pulmonary immunothrombosis [30].

### 4.4. Future Research Trends and Expectations

Reference burst analysis (Supporting Information 5: Figure S4) shows that as researchers deepen their understanding of the role of NETs in ALI/ARDS; they are committed to conducting therapeutic intervention studies for ALI/ARDS targeting NETs. Given that DNA is a major constituent of NETs, DNase I is deemed to be a practicable drug for getting rid of NETs. Recombinant human DNase‐I (rhDNase) has been shown to degrade NETs, thereby reducing inflammation around bronchi and blood vessels, and alleviating coagulopathy in ARDS [47]. However, due to the lack of precise and effective delivery strategies, targeting the delivery of DNase I to the inflamed lungs is very challenging, thus limiting the clinical application of DNase‐1 [48]. Thus, researchers will also prioritize the exploration of other new drugs with NETs as their therapeutic target in the future. As a new link between the innate and adaptive immune systems, the formation and activation of NETs can partially explain the reasons for the alleviation after the exacerbation of acute conditions and the persistence of chronic inflammation. Secondly, as a substance released by one of the immune cells, neutrophils, an appropriate number of NETs can capture and kill pathogens. Therefore, we expect that future researchers will be able to identify the optimal balance of NETs action in the human body and clarify the safety threshold of NETs under different disease states, which is of momentous consequence for the prevention and treatment of acute critical illnesses as well as chronic inflammation. In addition, although researchers have discovered some of the pathological symptoms produced by NETs in ALI/ARDS, some specific mechanisms are still waiting to be revealed, and different symptoms will have different mechanism pathways, so we hope that future researchers can develop highly specific drugs related to NETs.

## 5. Limitations

Although we collected data from the Embase, WOS, and PubMed databases, we did not cover all databases, such as Scopus, which may result in some relevant publications not being analyzed by us. Second, by limiting the language to English, we may overlook trends in non‐English articles. Moreover, as the citation analysis was conducted exclusively with WOS data, citation relationships recorded in other databases may have been overlooked, potentially compromising the comprehensiveness of the citation‐based findings. Last, as the relevant research is continuously being updated, recent publications require a certain amount of time to be fully cited, hence, the latest trends may be subject to some error.

## 6. Conclusions

This study employed bibliometric methods to deliver a thorough exposition of the research progress, hot spots as well as trends concerning NETs within ALI/ARDS. The first article related to this field was published in 2011, and since then, the number of publications has generally shown an upward trend. Both China and the United States boast a high ranking in the quantity of published articles and are regarded as exemplary representatives in the associated fields. Frontiers of Immunology ranks first in both publication volume and citation count, offering high reference value. The inflammatory response is a research hotspot in this field, while the relationship between NETs and thrombosis represents an emerging research hot spot. Additionally, NETs hold broad prospects as therapeutic targets and biomarkers for ALI/ARDS. The development of novel NETs‐targeting drugs to treat ALI/ARDS represents a future research trend.

## Consent

The authors have nothing to report.

## Disclosure

All authors have approved the submitted version of the manuscript and agreed to be accountable for any part of the work.

## Conflicts of Interest

The authors declare no conflicts of interest.

## Author Contributions

Yike Wang, Kang Zou, and Lingyan Zou made substantial contributions to the conception of the work. Zixin Luo made significant contributions to the data analysis and interpretation. Shaoyu Fu made significant contributions to the design of the work and the interpretation of data. Qianduo Li drafted the original manuscript. Zhiyuan Zhang and Nan Huang substantially contributed to the revision of the manuscript drafts.

## Funding

This work was supported by the Ganzhou Science and Technology Bureau, China (Grants 20222ZDX8182 and 2023LNS36644) and the Jiangxi Provincial Health Commission, China (Grant 202410348).

## Supporting Information

Additional supporting information can be found online in the Supporting Information section.

## Supporting information


**Supporting Information 1** Figure S1 Mapping on journals (A) and co‐cited journals (B) of studies concerning NETs in ALI/ARDS. In Figure A, the size of the nodes represents the number of publications, while in Figure B, it represents the number of co‐citations.

**Supporting Information 2** Figure S2 Author cooperative clustering view of studies concerning NETs in ALI/ARDS. The magnitude of the nodes stands for the publication volume, while the thickness of the lines concerning the nodes demonstrates the intensity of cooperation between two nodes.

**Supporting Information 3** Table S1 The 10 most frequently co‐cited references. Table S2 The main contents of the top 10 strong citation outburst references.

**Supporting Information 4** Figure S3 Mapping on co‐cited references of studies concerning NETs in ALI/ARDS.

**Supporting Information 5** Figure S4 Top 10 strong citation outbreaks of references.

## Data Availability

The data that support the findings of this study are available on request from the corresponding author. The data are not publicly available due to privacy or ethical restrictions.
