# Supplementary figures and images for: Emerging Topics and Trends in Neutrophil Extracellular Traps in ARDS: A Bibliometric and Visual Analysis
Source: Mediators Inflamm. 2025 Dec 29;2025:1015955. doi: 10.1155/mi/1015955 (PMC12767411; doi:10.1155/mi/1015955)

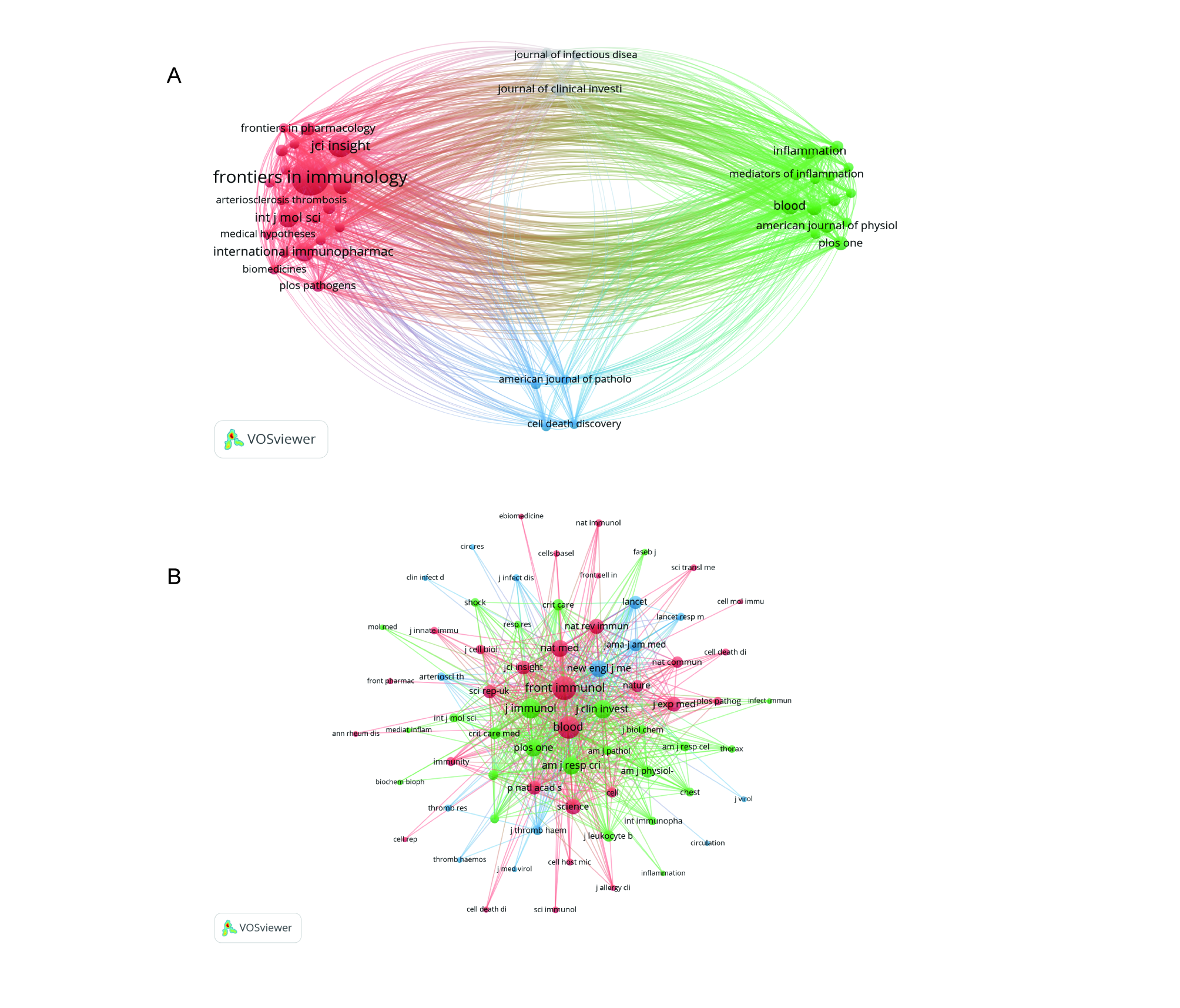

Supplement: Supplementary file 1 — Supporting Information 1 Figure S1 Mapping on journals (A) and co‐cited journals (B) of studies concerning NETs in ALI/ARDS. In Figure A, the size of the nodes represents the number of publications, while in Figure B, it represents the number of co‐citations. [file MI-2025-1015955-s001.tif]

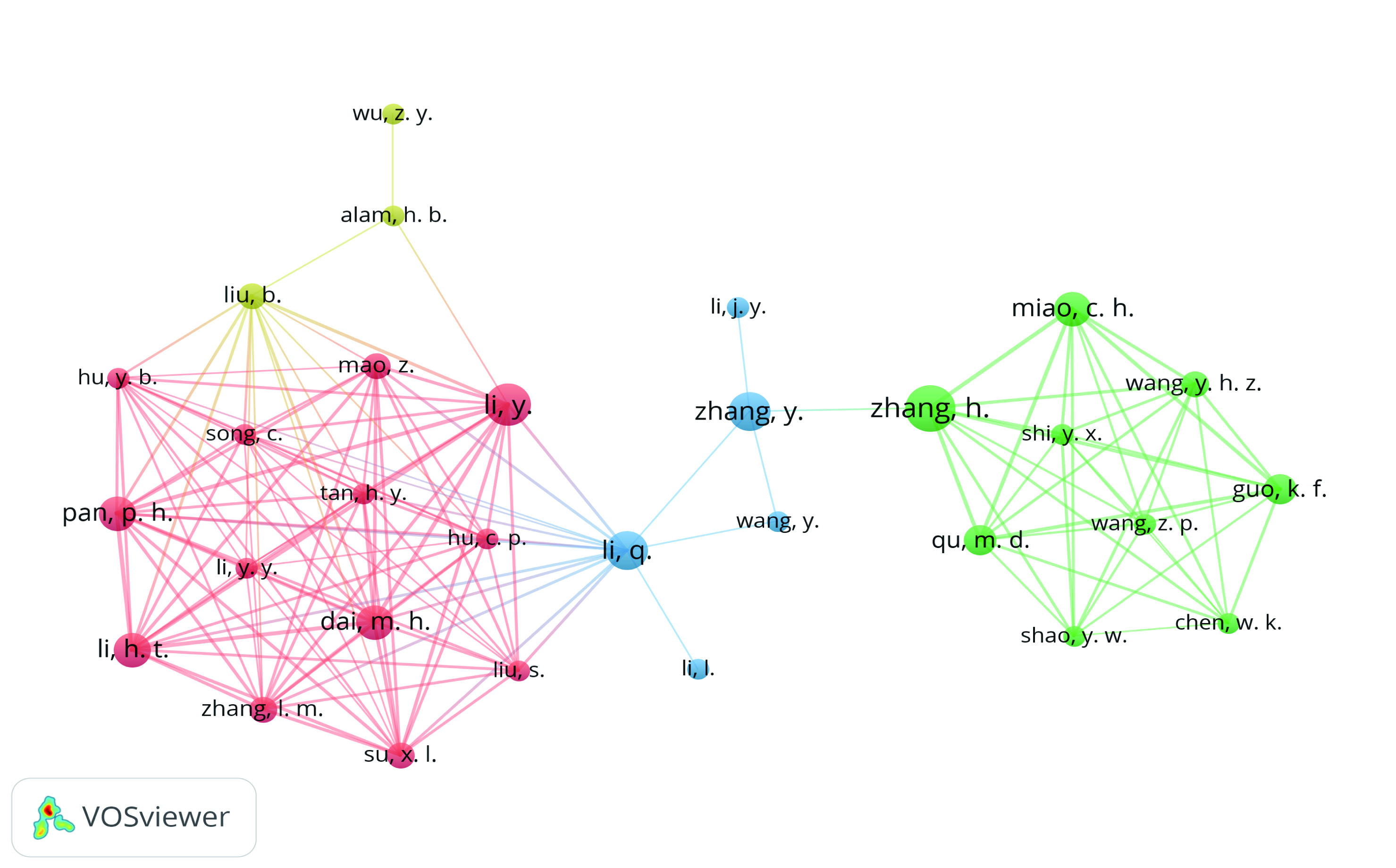

Supplement: Supplementary file 2 — Supporting Information 2 Figure S2 Author cooperative clustering view of studies concerning NETs in ALI/ARDS. The magnitude of the nodes stands for the publication volume, while the thickness of the lines concerning the nodes demonstrates the intensity of cooperation between two nodes. [file MI-2025-1015955-s002.tif]

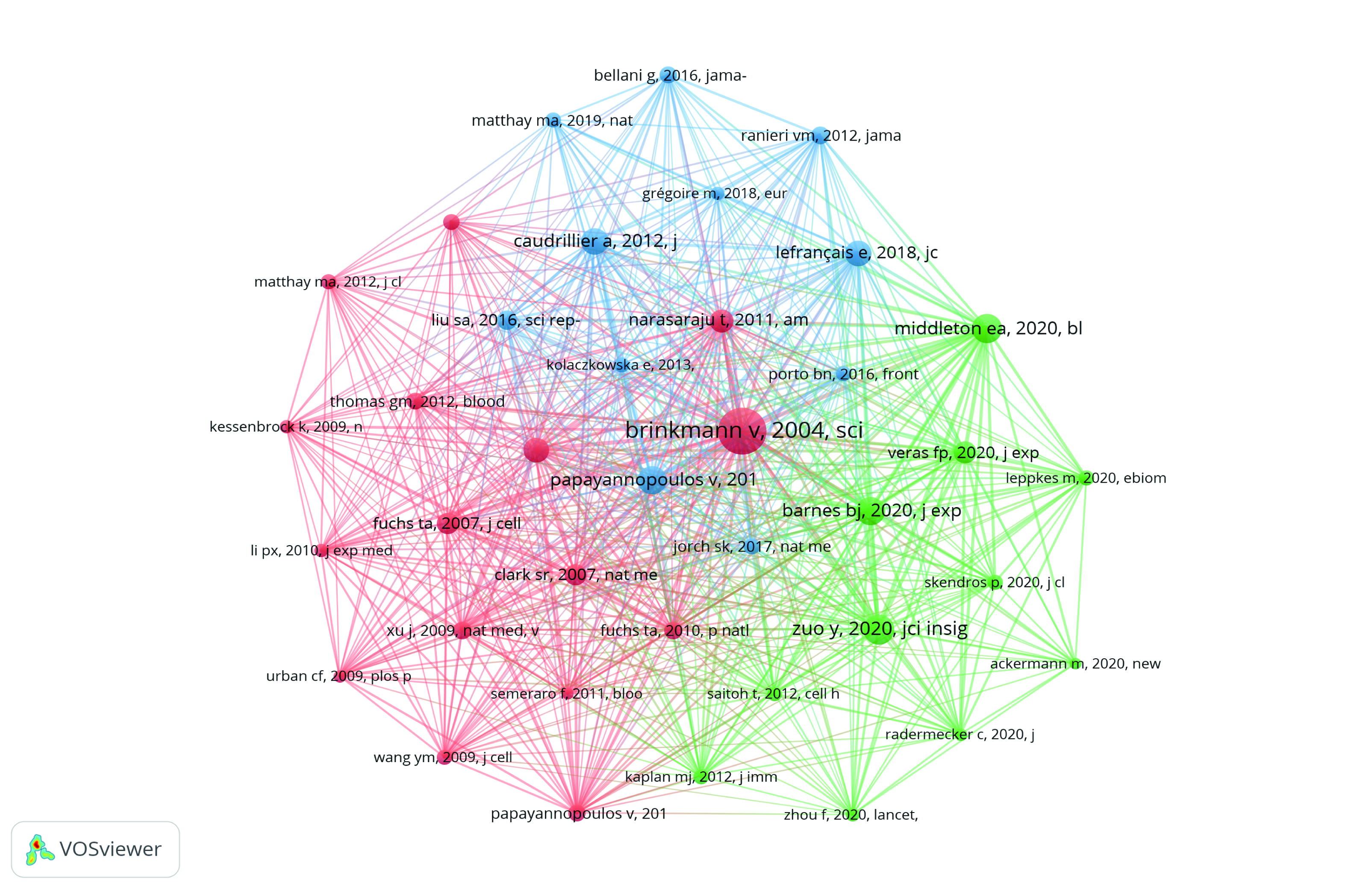

Supplement: Supplementary file 3 — Supporting Information 3 Table S1 The 10 most frequently co‐cited references. Table S2 The main contents of the top 10 strong citation outburst references. [file MI-2025-1015955-s003.tif]

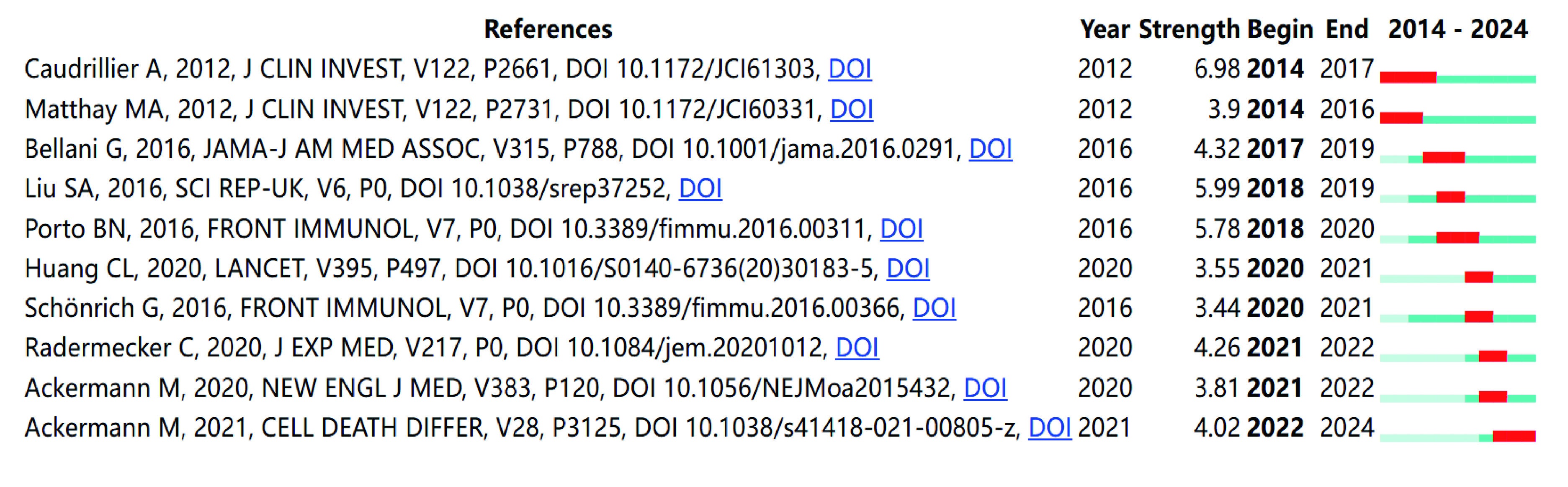

Supplement: Supplementary file 4 — Supporting Information 4 Figure S3 Mapping on co‐cited references of studies concerning NETs in ALI/ARDS. [file MI-2025-1015955-s004.tif]
