# Supplementary material for: Emerging Topics and Trends in Neutrophil Extracellular Traps in ARDS: A Bibliometric and Visual Analysis
Source: Mediators Inflamm. 2025 Dec 29;2025:1015955. doi: 10.1155/mi/1015955 (PMC12767411; doi:10.1155/mi/1015955)
Supplement: Supplementary file 5 — Supporting Information 5 Figure S4 Top 10 strong citation outbreaks of references. [file MI-2025-1015955-s005.docx]

| Rank | Co-cited Reference | Citations |
| --- | --- | --- |
| 1 | BRINKMANN V, 2004, SCIENCE, V303, P1532, DOI 10.1126/SCIENCE.1092385 | 129 |
| 2 | ZUO Y, 2020, JCI INSIGHT, V5, DOI 10.1172/JCI.INSIGHT.138999 | 70 |
| 3 | MIDDLETON EA, 2020, BLOOD, V136, P1169, DOI 10.1182/BLOOD.2020007008 | 67 |
| 4 | BARNES BJ, 2020, J EXP MED, V217, DOI 10.1084/JEM.20200652 | 66 |
| 5 | PAPAYANNOPOULOS V, 2018, NAT REV IMMUNOL, V18, P134, DOI 10.1038/NRI.2017.105 | 65 |
| 6 | CAUDRILLIER A, 2012, J CLIN INVEST, V122, P2661, DOI 10.1172/JCI61303 | 60 |
| 7 | LEFRANÇAIS E, 2018, JCI INSIGHT, V3, DOI 10.1172/JCI.INSIGHT.98178 | 57 |
| 8 | SAFFARZADEH M, 2012, PLOS ONE, V7, DOI 10.1371/JOURNAL.PONE.0032366 | 54 |
| 9 | NARASARAJU T, 2011, AM J PATHOL, V179, P199, DOI 10.1016/J.AJPATH.2011.03.013 | 50 |
| 10 | VERAS FP, 2020, J EXP MED, V217, DOI 10.1084/JEM.20201129 | 46 |

**Table S1 The 10 most frequently co-cited references**

**Table S2 The main contents of the top 10 strong citation outburst references**

| Rank | Strength | Research Content |
| --- | --- | --- |
| 1 | 6.98 | The formation of NETs is induced by activated platelets in transfusion-related acute lung injury (TRALI) (12). |
| 2 | 3.9 | A variety of clinical conditions can induce ARDS, including pneumonia, sepsis, aspiration of gastric contents, and severe trauma (32). |
| 3 | 4.32 | Epidemiology, nursing patterns, and mortality among patients with ARDS in intensive care units (33). |
| 4 | 5.99 | NETs are indirectly triggered by lipopolysaccharide (LPS) and give rise to ALI, with DNase I potentially serving as a new adjunctive therapy for ALI (34). |
| 5 | 5.78 | Summarizes the latest NETs knowledge in pulmonary diseases and methods to regulate their harmful effects (35). |
| 6 | 3.55 | Infection with 2019-nCoV lead to clustering of severe respiratory diseases resembling SARS-CoV ones and links to ICU admissions and high mortality (36). |
| 7 | 3.44 | Discussed the mechanisms for the generation of NETs during viral infections, and also elucidated the viral immune evasion mechanisms that target NETs (37). |
| 8 | 4.26 | In severe COVID-19, NETs infiltrate the pulmonary airways, interstitium, and vascular compartments (38). |
| 9 | 3.81 | Analyze pulmonary vasculitis, thrombosis, and angiogenesis in COVID-19 patients (39). |
| 10 | 4.02 | A proposed mechanism by which NETs promote tissue damage and immunothrombosis in COVID-19 patients has been put forward (40). |
